# Socioeconomic status in life course is associated with dental appearance dissatisfaction

**DOI:** 10.1590/1807-3107bor-2024.vol38.0051

**Published:** 2024-06-24

**Authors:** Luiz Alexandre CHISINI, Fabiana VARGAS-FERREIRA, Giulia Tarquinio DEMARCO, Karen Glazer PERES, Marco Aurélio PERES, Bernardo Lessa HORTA, Flávio Fernando DEMARCO

**Affiliations:** (a) Universidade Federal de Pelotas – UFPel, Graduate Program in Dentistry, Pelotas, RS, Brazil.; (b) Universidade Federal de Minas Gerais – UFMG, School of Dentistry, Department of Social and Preventive Dentistry, Belo Horizonte, MG, Brazil.; (c) National Dental Research Institute Singapore, National Dental Centre Singapore; Oral Health ACP, Health Services and Systems Research Programme, Duke-NUS Medical School, Singapore.; (d) National Dental Centre Singapore, Dental Research Institute Singapore, Singapure.; (e) Universidade Federal de Pelotas – UFPel, Graduate Program in Epidemiology, Pelotas, RS, Brazil.; (f) Universidade Federal de Pelotas – UFPel, Graduate Program in Dentistry and Epidemiology, Pelotas, RS, Brazil.

**Keywords:** Income, Social Class, Malocclusion, Health Inequities, Esthetics, Dental Caries

## Abstract

The present study aimed to investigate the prevalence of dissatisfaction with dental appearance among 24-year-old Brazilian adults and the associated factors in life course. A subsample (n = 720) of the 1982 Pelotas Birth Cohort in southern Brazil was investigated at the ages of 15 and 24 years using clinical (caries and periodontal) examinations and interviews. The outcome was dissatisfaction with dental appearance at the age of 24 years. Covariate variables included socioeconomic factors, oral health, and dissatisfaction with general appearance collected during different periods of life. Poisson regression models with robust variance were applied. The prevalence of dissatisfaction with dental appearance was 43.5% (95%CI: 39.8–47.1). Individuals with downward income mobility (PR = 1.22, 95%CI: 1.07–1.79) and those always poor (PR = 1.21, 95%CI: 1.00–1.57) presented a higher prevalence of dissatisfaction with their dental appearance even after oral health variables and dissatisfaction with general appearance were controlled for. Moderate/severe malocclusion at 15 years (PR = 1.34, 95%CI: 1.13–1.59), highest experience of untreated dental caries at 24 years (PR = 1.82, 95%CI: 1.46–2.27), and dental pain experience at 24 years (PR = 1.29, 95%CI: 1.22–1.75) were associated with the outcome. Also, the prevalence of dissatisfaction with dental appearance was 20% higher (PR = 1.20, 95%CI: 1.01–1.43) among those dissatisfied with their general appearance. Our findings demonstrated a high prevalence of dissatisfaction with dental appearance among young adults. Lifetime economic disadvantage and dental problems (malocclusion at 15 years, untreated dental caries at 24 years, and dental pain at 24 years) were associated with dissatisfaction with dental appearance among young adults.

## Introduction

Dental appearance is an important feature in determining facial attractiveness and can vary according to different cultures and their respective socioeconomic development.^1,2^ However, there is a cross-cultural consensus that the smile is a fundamental element of physical attractiveness.^2^ A beautiful smile can be correlated with improved self-esteem^3^ and good social interactions,^4^ playing a fundamental role in the social life context. People with aligned and whiter teeth have also been considered wealthier and more intelligent.^5^ Thus, individuals who underwent bleaching treatments had an improved quality of life.^6^

On the other hand, unaesthetic dental appearance can affect social interactions.^1^ It is associated with an increase in the prevalence of bullying or cyberbullying among children, leading to physical, social, and psychological impairment in their lives.^7,8^ In adulthood, dental appearance may lead to discrimination episodes,^9^ and the risk could be higher among females,^10^ demonstrating an underlying gender component. Therefore, dental appearance comprises an important aspect of oral health and oral health-related quality of life (OHRQoL).^1, 11^

The prevalence of dissatisfaction with dental appearance ranges from 8.1 to 52.8%, according to different countries and ages.^12-20^ Studies have found that dissatisfaction with dental appearance is associated with worse OHRQoL,^11^ and this could be mainly due to malocclusion.^12-15,20^ The relationship between individuals’ socioeconomic position and health is well known, and social mobility predicts important oral health outcomes in early adulthood.^21^ However, few studies have explored the underlying socioeconomic factors that could be related to the perception of dental appearance.

To the best of our knowledge, no longitudinal population-based study has been carried out following adolescents to adulthood, investigating the relationships between dissatisfaction with dental appearance, sociodemographic factors, and oral conditions. Therefore, the objectives of this study were to a) estimate the prevalence of dissatisfaction with dental appearance in a population-based sample; and b) investigate the associated factors with dental appearance dissatisfaction. We hypothesized that poverty in the life course may lead to an increase in dental appearance dissatisfaction.

## Methodology

This study was reported following the Strengthening the Reporting of Observational Studies in Epidemiology (STROBE) statement.

### Data source

The study was carried out in Pelotas, a town located in southern Brazil. Pelotas has an estimated population of 343,132 inhabitants and a Human Development Index of 0.739. The water fluoridation scheme was implemented in 1962, covering almost all of the urban population.

In 1982, all the infants born at three local maternity hospitals were identified (n = 5,914). The 5,914 liveborn babies were measured, and their mothers were interviewed at birth and in several follow-up visits. Detailed information about the methods of this cohort study is available elsewhere.^22^ In 1997, a cross-sectional oral health study was conducted (OHS-97), and a sample of 900 individuals was selected (when the participants were 15 years old). The OHS-97 consisted of an interview containing questions about oral hygiene habits, dental service utilization, and dental examinations to assess the presence of dental caries, malocclusion, and periodontal diseases in adolescents. The 888 adolescents (98.7%) evaluated in the OHS-97 were assessed again in 2006 (OHS-06). They were interviewed and examined for oral health conditions, such as dental caries and periodontal status. All individuals from the OHS-97 sample were considered eligible for the present study. Individuals not selected in the OHS-97 were excluded from the sample. All the dental examinations were carried out at the participants’ homes (preferably in a specifically designated room) using personal protective equipment (PPE). Dental examinations were performed by 10 calibrated dentists, and questionnaires were administered by seven trained interviewers. We tested the questionnaire and performed the training and calibration process in a similarly aged population not eligible for the study.

The interviewers were trained and calibrated in a population of similar age to that of the sample prior to the fieldwork. Dental clinical examinations were carried out by dentists with visual inspection and headlight using a probe (Community Periodontal Index probes - CPI) and mirror, and the surface of the participants’ teeth was dried with gauze pads.^23^ Inter- and intra-examiner reliability of OHS-06 was calculated using kappa statistics for dental caries and the malocclusion classification. The lowest kappa value was 0.65 for dental caries and 0.60 for malocclusion.

### Outcome variables

The outcome of the present study was dissatisfaction with dental appearance at the age of 24 years, collected using the following question: “Are you satisfied with the appearance of your teeth?“ (yes, no).^12^

### Exploratory variables

Exploratory variables were collected during different assessments. Sex and maternal schooling were collected at birth. Maternal schooling was specified in years of study and categorized as a) ≥ 12; b) 9 to 11; c) 5 to 8; and d) 0 to 4.

Family income was collected in 1982 based on five categories of Brazilian minimum wages (< 1, 1–3, 3.1–6, 6.1–10, and > 10). As income at birth was initially collected in five categories, we regrouped the categories to classify income families in tertiles, as follows: a principal component analysis was carried out for four variables strongly related to wealth in our sample: delivery care payment mode (out-of-pocket, public free, or private health insurance) and mother’s schooling, height, and skin color.

The first component originated as a number used to rank individuals according to family income groups. The cut-off points were found within each category so that three nearly equal-sized groups were achieved. Family income was also collected when individuals were 19 years old (in 2001). Tertiles were generated from the sum of the reported values. A total of nine groups were created. These groups represented all possible combinations of the tertiles at birth and at 19 years of age. The highest and the middle family income tertiles were combined in a single group referred to as ‘non-poor’, aiming at increasing the statistical analysis power. In contrast, the lowest tertile is referred to as ‘poor’. This organization resulted in four possible categories: a) never poor; b) downward mobile; c) upward mobile; and d) always poor. ^24^

Occlusal status was collected by clinical examination at the age of 15 years according to the Dental Aesthetic Index (DAI) using a periodontal probe:^23^ (i) normal or minor malocclusion (minimal or no orthodontic treatment required, ≤ 25); (ii) definite malocclusion (optional orthodontic treatment, 26 to 30); (iii) severe malocclusion (highly desirable orthodontic correction, 31 to 35); and (iv) very severe malocclusion (mandatory orthodontic correction, >36). Occlusal status was dichotomized into: a) normal occlusion/mild malocclusion and b) moderate/severe/very severe malocclusion.^12, 23^ The use of orthodontic appliance in some moment of life was assessed at 24 years: “Have you ever used orthodontic appliances at some point in your life?” (yes/no).

Self-reported skin color at the age of 24 years was collected according to the Brazilian Institute of Geography and Statistics (IBGE) under the following categories: white, yellow, American native, brown, and black,^25^ and grouped into a) white and Asian; b) brown; and c) black. Dental service use in the last year (“Have you visited a dentist in the last 12 months?”) and dental pain in the last month (“Have you had toothache in the last four weeks?”) were collected at 24 years of age. Also, a question about general appearance satisfaction was asked: “Are you satisfied with your appearance? “(yes, no).

Dental caries was assessed by DMFT (decayed, missing, and filled teeth) index^23^ at 24 years. Untreated dental caries was estimated by the “D” component of the DMFT index and divided into tertiles. Periodontal examinations comprised full-mouth probing at six sites per tooth (mesiobuccal, midbuccal, distobuccal, distolingual, midlingual, and mesiolingual) using a PCP2 probe. All sites were probed, and gingival bleeding was assessed after 10 seconds to verify the presence/absence of gingival bleeding. The variable was categorized as absent (no bleeding) or present when one or more teeth had gingival bleeding.^26, 27^ Probing was performed on all sites for the detection of calculus . This variable was divided into tertiles.^26, 27^ All sites were probed to evaluate periodontal pockets, and the pocket should have a probing depth of ≥4 mm on at least one site. The variable was dichotomized into absence or presence of a periodontal pocket. Presence was considered if periodontal pockets were present on at least one surface.^26, 27^

### Statistical analyses

Data were analyzed using STATA software, version 16.0 (Stata Corp., College Station, TX, USA). Descriptive analysis was performed to assess the prevalence of the independent and dependent variables, 95% confidence intervals (95% CI), and the response rate of each variable. Poisson regression models with robust variance were applied for prevalence ratio estimates. All variables with p<0.20 in the bivariate analysis were included in the multivariate analysis. Only variables with p≤0.05 were maintained in the final model.

### Ethical issues

Consent for interviews and exams were obtained, and both projects (at the ages of 15 and 24 years) were approved by the Ethics Committee of the Federal University of Pelotas. Adolescents who presented dental-treatment needs were referred to the Dental Clinic of the Graduate Program in Dentistry at the Federal University of Pelotas.

## Results

A total of 720 individuals were examined (response rate of 80% of OHS-97). The main reason for non-response was family refusal. Data from OHS-97 were similar to those of the original cohort concerning sex and maternal schooling (Table 1). Around half of the individuals were male (52.6%). A total of 32.0% showed prevalence of moderate or severe malocclusion (95%CI: 28.5–35.4%) and 23.0% reported dental pain in the last month at the age of 24 years (95%CI: 20.0–26.3). The mean DMFT index was 5.6 (standard deviation [SD] = 4.1), and the mean of untreated dental caries was 2.9 (SD = 3.1). The prevalence of dissatisfaction with dental appearance was 43.5% (95% CI: 39.8–47.1) (Table 1).

**Table 1:** Description of socioeconomic, demographic, and oral health characteristics of participants in the 1982 Pelotas Birth Cohort. Pelotas, RS, Brazil (n = 720 individuals).

Variable/Category	n (%)	95%CI	Response rate	Original cohort* (n = 5,914)
Sex			100.0	
Male	379 (52.6)	48.9–56.3		3,037 (51.4)
Female	341 (47.4)	43.6–51.0		2,876 (48.6)
Skin Color			99.9	
White and Asiatic	559 (77.7)	74.5–80.7		
Brown	55 (7.6)	5.8–09.8		
Black	105 (14.7)	12.0–17.3		
Maternal schooling at birth (years)			99.7	
≥ 12	88 (12.3)	09.9–14.8		839 (14.2)
9 to 11	75 (10.4)	08.2–12.8		654 (11.1)
5 to 8	321 (44.7)	41.0–48.4		2,454 (41.6)
0 to 4	234 (32.6)	29.2–36.1		1,960 (33.2)
Family income change			100.0	
Never poor	332 (46.1)	42.4–49.8		
Downward mobile	131 (18.2)	15.4–21.2		
Upward mobile	94 (13.1)	10.6–15.7		
Always poor	163 (22.6)	19.6–25.8		
Occlusal status (15y)			98.6	
Normal occlusion/mild malocclusion	483 (68.0)	64.4–71.4		
Moderate/severe malocclusion/very severe	227 (32.0)	28.5–35.4		
Use of orthodontic appliance (24y)			100.0	
No	671 (93.2)	91.1–94.9		
Yes	49 (6.8)	05.0–08.8		
Dental service use in the last year (24y)			96.0	
No	307 (44.4)	40.0–48.1		
Yes	384 (55.6)	51.8–59.2		
Dental pain (24y)			99.0	
No	549 (77.0)	73.6–79.9		
Yes	164 (23.0)	20.0–26.3		
Untreated dental caries (tertiles - 24y)			100.0	
Lower (1^st^)	365 (50.7)	46.9–54.4		
Intermediate (2^nd^)	137 (19.0)	16.2–22.0		
Highest (3^rd^)	218 (30.3)	26.9–33.7		
Gingivitis (24y)			100.0	
Yes	450 (62.5)	58.8–66.0		
No	270 (37.5)	33.9–41.1		
Dental calculus (tertile - 24y)			100.0	
1^st^ tertile (≤ 15,38%)	248 (34.4)	30.9–38.0		
2^nd^ tertile (15,79 to 34.62%)	233 (32.4)	28.9–35.9		
3^rd^ tertile (≥34.78%)	239 (33.2)	29.7–36.7		
Periodontal pockets (24y)			100.0	
No	696 (96.7)	95.0–97.8		
Yes	24 (3.3)	02.1–04.7		
Dissatisfaction with appearance (24y)			100.0	
No	449 (62.4)	58.7–65.9		
Yes	271 (37.6)	34.1–41.2		
Dissatisfaction with dental appearance (24y)			96.7	
No	393 (56.5)	52.8–60.1		
Yes	303 (43.5)	39.8–47.1		

The unadjusted and adjusted prevalence ratios for dissatisfaction with dental appearance according to covariables are displayed in Table 2. In the adjusted model, an association between dissatisfaction with dental appearance and family income change was observed: individuals with downward income mobility (PR = 1.22, 95%CI: 1.07–1.79) and those always poor (PR = 1.21, 95%CI: 1.00–1.57) presented a higher prevalence of dissatisfaction with their dental appearance even after exploratory variables (sex, skin color, maternal schooling at birth, oral health variables, and dissatisfaction with general appearance) were controlled for. Individuals with moderate/severe malocclusion (PR = 1.34, 95%CI: 1.13–1.59), high experience of untreated dental caries (PR = 1.82, 95%CI: 1.46–2.27), and dental pain experience (PR = 1.29, 95%CI: 1.22–1.75) were associated with higher prevalence of dental appearance dissatisfaction. Dissatisfaction with the general appearance was associated with a 20% higher prevalence of dissatisfaction with dental appearance (PR = 1.20, 95%CI: 1.01–1.43).

**Table 2:** Unadjusted (c) and adjusted (a) prevalence ratios (PR) for dissatisfaction with dental appearance in a sample of individuals according to covariables. Pelotas, RS, Brazil (n = 720 individuals).

Variable/Category	Crude mode	p-value	Adjusted model	p-value
	
	PR^a^ (95%CI)		PR^a^ (95%CI)	
Sex		0.371		-
Male	1		-	
Female	1.08 (0.91–1.28)			
Skin Color		0.019		-
White and Asiatic	1		-	
Brown	1.04 (0.74–1.44)			
Black	1.29 (1.05–1.59)			
Maternal schooling at birth (years)		0.013		-
≥ 12	1		-	
9 to 11	1.57 (1.05–2.35)			
5 to 8	1.35 (0.95–1.95)			
0 to 4	1.62 (1.14–2.31)			
Family income change		0.006		0.040
Never poor	1		1	
Downward mobile	1.42 (1.13–1.77)		1.22 (1.07–1.79)	
Upward mobile	1.34 (1.04–1.73)		1.30 (0.94–1.78)	
Always poor	1.28 (1.03–1.61)		1.21 (1.00–1.57)	
Occlusal status (15y)		< 0.001		< 0.001
Normal occlusion/mild malocclusion	1		1	
Moderate/severe malocclusion/very severe	1.44 (1.22–1.70)		1.34 (1.13–1.59)	
Use of orthodontic appliance (24y)		0.820	-	-
No	1			
Yes	0.94 (0.56–1.58)			
Dental service use in the last year (24y)		0.875	-	-
No	1			
Yes	1.01 (0.85–1.21)			
Dental pain (24y)		< 0.001		< 0.001
No	1		1	
Yes	1.47 (1.24–1.74)		1.29 (1.22–1.75)	
Untreated dental caries (tertiles - 24y)		< 0.001		< 0.001
Lower (1^st^)	1		1	
Intermediate (2^nd^)	1.33 (1.03–1.71)		1.23 (0.97–1.72)	
Highest (3^rd^)	2.03 (1.68–2.44)		1.82 (1.46–2.27)	
Gingivitis (24y)		0.782		-
Yes	1		-	
No	0.97 (0.81–1.16)			
Dental calculus (tertile - 24y)		0.031		-
1^st^ tertile (≤ 15,38%)	1		-	
2^nd^ tertile (15,79 to 34.62%)	1.02 (0.82–1.27)			
3^rd^ tertile (≥34.78%)	1.25 (1.02–1.53)			
Periodontal pockets (24y)		0.564		-
No	1		-	
Yes	0.86 (0.51–1.44)			
Dissatisfaction with appearance (24y)		0.047		0.048
No	1		1	
Yes	1.21 (1.03–1.42)		1.20 (1.01–1.43)	

## Discussion

This study found a high prevalence (43.53%) of dissatisfaction with dental appearance in a sample of adults and confirms the hypothesis that poverty in the life course was associated with increased dental appearance dissatisfaction. Individuals at the age of 24 years dissatisfied with their dental appearance were more likely to be always poor or have downward social mobility, have malocclusion at the age of 15 years, have dental pain, and untreated dental caries at the age of 24 years, and dissatisfied with their general appearance.

A high prevalence of dissatisfaction with dental appearance was found in the present sample, similar to the highest prevalence reported in the literature.^12-20^ A probable explanation for such difference is the elevated desire for aesthetic dental procedures observed in the Brazilian population.^28,29^ A study carried out with university students found that 74.4% of the sample desired to undergo aesthetic dental treatments to improve their smile.^28^ Similarly, a population-based study observed that 85.9% of individuals desired to have their teeth bleached to improve their appearance.^29^ Aesthetic demand in Brazil is high and can be observed in all aspects of individual life. Thus, comparisons with high aesthetic standards, often unattainable, could increase dissatisfaction with their appearance, reflecting the high number of performed plastic surgeries^30^ and concerns regarding general aesthetics could have an impact on dental aesthetics given that the smile is a fundamental component of individuals’ appearance and affects OHQoL.^1,11^ This finding highlights the association between general and dental dissatisfaction in the present sample. Also, the high prevalence observed in the present study could be influenced by the age at which the outcome was assessed. Young adults have higher social interactions and tend to give greater importance to aesthetic aspects than do other age groups.

Some studies, mainly those with a cross-sectional design, have found associations between malocclusion and dissatisfaction with dental appearance .^13-15^ In this context, our findings, using a longitudinal design, corroborate previous observations. We observed that adolescents with moderate/severe malocclusion at the age of 15 years presented a 34% higher prevalence of dissatisfaction with their dental appearance at 24 years, even after controlling for socioeconomic and oral health covariables. Actually, individuals with untreated malocclusion present a greater risk of bullying^7, 8^ and more reported episodes of discrimination than those with aligned teeth.^10^ This can lead to an impact on the OHRQoL,^1,11^ which can explain the association between malocclusion and dissatisfaction with dental appearance observed in our study. Therefore, the presence of inappropriately treated malocclusion in adolescents could negatively impact individuals’ dental satisfaction,^16, 31^ even among young adults. There is a growing concern related to misaligned teeth and dental appearance in Brazil that has led to a greater demand for orthodontic treatments, even by adults.^32,33^

Moreover, individuals with the highest tertile of untreated dental caries and those who experienced dental pain were strongly associated with dental appearance dissatisfaction, which is in agreement with most studies, showing that negative clinical dental conditions are associated with poorly perceived dental appearance of oral health.^28,29^ The presence of carious lesions, especially in anterior teeth, can compromise the individual’s perceptions of their aesthetics, reducing self-esteem and even compromising individuals’ relationships and OHRQoL.^34^

The association of income change throughout life with dissatisfaction with dental appearance was an important finding of our study. Individuals with downward social mobility and those who were always poor remained associated with higher dissatisfaction with dental appearance in the model even when adjusted for oral health status (i.e., untreated dental caries, dental pain, and malocclusion), demonstrating that socioeconomic factors have underlying influences on the perception of dental aesthetics. Evidence suggests that the socioeconomic trajectory is a dynamic way of monitoring and evaluating the effects of changes in socioeconomic levels on the health status of the population.^21^ Consistent findings ratify that individuals experiencing poverty trajectory during their life course were more likely to have a worse evaluation of their oral health status.^35-38^ Potential discrimination amongst socially marginalized groups due to dental appearance was observed among Canadians.^9^ In another study with Brazilian adolescents, negative self-reports were associated with lower family income.^16^ The perception of oral health (dental appearance) is directly affected by socioeconomic factors^9^ and could influence oral health decisions and patterns of healthcare utilization.^39^ It is well established that individuals from lower socioeconomic status are more exposed to various risk factors that could affect self-perception of oral health and well-being.^40^

Important points must be highlighted in this study. The present sample is a population-based study nested in a birth cohort study and presents a high response rate, practically eliminating the possibility of selection bias. This study has good internal validity due to all procedures of training and calibration performed. A multidisciplinary cohort design allows for a precise collection of behavioral and socioeconomic variables in several moments of the life course. The variables included in the present study have few losses, and dental visit in the last year is the variable with the highest losses (4.0%). However, this study presents some limitations that should be highlighted. The generalizability of the present results should be limited to the population with similar characteristics in Brazil. Although the present study was performed with a representative sample of individuals from Pelotas, the results should not be extrapolated to Brazil as a whole. Brazil has important regional differences in terms of ethnicity, income, and use of dental services. Moreover, the use of a single question as the outcome measure could be considered a limitation because the question may not fully capture the complexity of the underlying construct. This may limit the depth of insight into the multifaceted nature of the phenomenon under investigation.

Our results highlight the importance of considering socioeconomic factors in the development of public policies related to oral health and aesthetics. Policies aimed at reducing income inequality and providing access to affordable dental care could potentially mitigate the prevalence of dissatisfaction with dental appearance. Additionally, focusing on preventive measures and early intervention for malocclusion and dental caries could help improve oral health and satisfaction with dental appearance, leading to improvement in individuals’ OHRQoL.

## Conclusion

Our findings demonstrated a high prevalence of dissatisfaction with dental appearance in young adults in this Brazilian birth cohort. Poverty trajectories were associated with dissatisfaction with dental appearance even after controlling for oral health variables. Clinical conditions (presence of malocclusion, untreated caries, and dental pain) were also related to dental appearance dissatisfaction. Individuals dissatisfied with general appearance were more dissatisfied with dental appearance.
